# Neuronal ApoE4 in Alzheimer’s disease and potential therapeutic targets

**DOI:** 10.3389/fnagi.2023.1199434

**Published:** 2023-06-02

**Authors:** Lan Zhang, Yiyuan Xia, Yuran Gui

**Affiliations:** School of Medicine, Jianghan University, Wuhan, Hubei, China

**Keywords:** Alzheimer’s disease, apolipoprotein E4, Aβ, tau, inflammation

## Abstract

The most prevalent genetic risk factor for Alzheimer’s disease (AD) is Apolipoprotein E (ApoE), a gene located on chromosome 19 that encodes three alleles (e2, e3, and e4) that give rise to the ApoE subtypes E2, E3, and E4, respectively. E2 and E4 have been linked to increased plasma triglyceride concentrations and are known to play a critical role in lipoprotein metabolism. The prominent pathological features of AD mainly include senile plaques formed by amyloid β (Aβ_42_) aggregation and neuronal fibrous tangles (NFTs), and the deposited plaques are mainly composed of Aβ hyperphosphorylation and truncated head. In the central nervous system, the ApoE protein is primarily derived from astrocytes, but ApoE is also produced when neurons are stressed or affected by certain stress, injury, and aging conditions. ApoE4 in neurons induces Aβ and tau protein pathologies, leading to neuroinflammation and neuronal damage, impairing learning and memory functions. However, how neuronal ApoE4 mediates AD pathology remains unclear. Recent studies have shown that neuronal ApoE4 may lead to greater neurotoxicity, which increases the risk of AD development. This review focuses on the pathophysiology of neuronal ApoE4 and explains how neuronal ApoE4 mediates Aβ deposition, pathological mechanisms of tau protein hyperphosphorylation, and potential therapeutic targets.

## 1. Introduction

**Figure 1:**
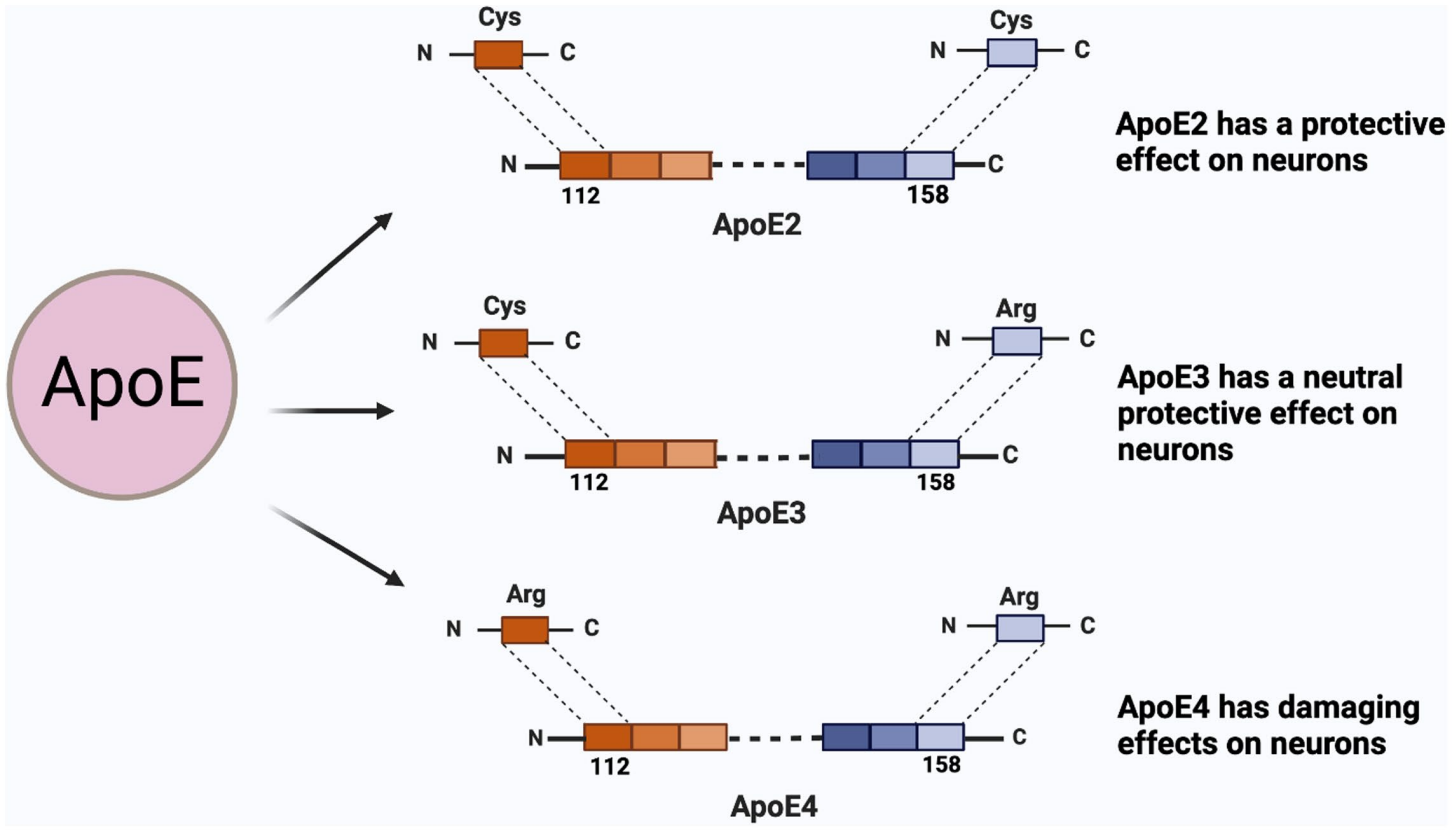
ApoE isoforms and their functions on neuron. ApoE has three isoforms, namely E2, E3, E4; The difference between the three are the 112-position and the 158-position amino acid. ApoE2 is cysteine at both sites and presents a protective effect on neurons; The 112th position of ApoE3 is cysteine, and 158 is arginine, which protects neurons between E2 and E4; ApoE4 is arginine at both sites.

**Figure 2:**
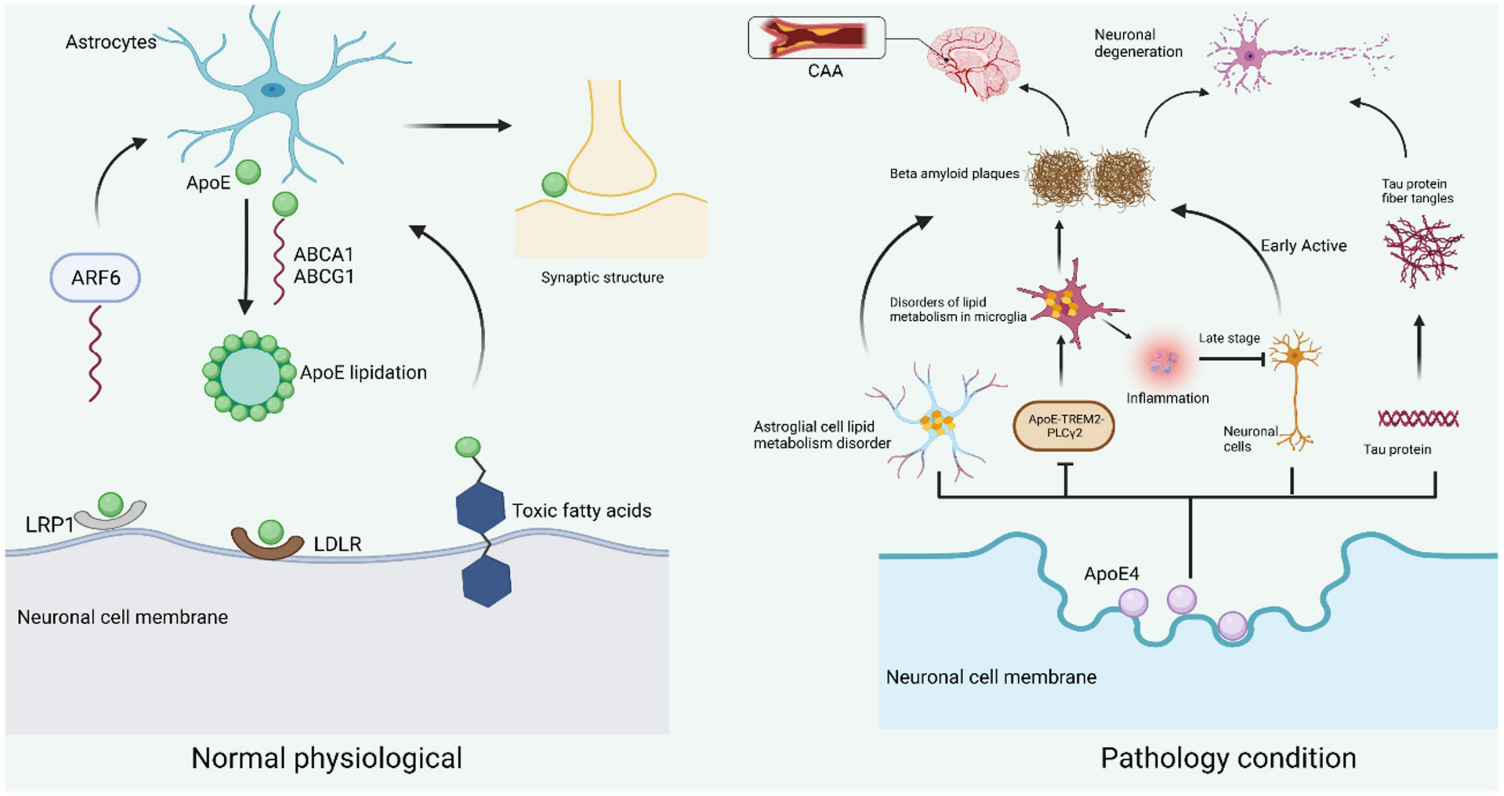
Neuronal ApoE function in physiological and pathological states. Under normal physiological conditions, ApoE is mainly produced in astrocytes and is transported by the ATP-binding cassette transporters ABCA1 and ABCG1 for lipidation and involved in lipid metabolism. ABCA1 will be recycled into astrocytes by ARF6. On the surface of neuronal cell membranes, LRP1 and LDLR are the two main receptors for ApoE. By binding to the receptors, they exert their physiological functions in neurons, including neuronal repair, cell membrane stability, dendrite growth, and maintenance of synaptic plasticity (left). When neurons are subjected to pathological conditions such as injury, stress, and aging, ApoE4 mainly originates from neuronal cells and causes impaired lipid metabolism in astrocytes, resulting in reduced Aβ protein clearance; ApoE4 also leads to lipid accumulation in microglia by inhibiting the ApoE-TREM2-PLCγ2 signaling pathway, thus inducing inflammation and Aβ protein deposition. ApoE4 also causes hyperphosphorylation of Tau protein to form fibrillary tangles, and Aβ amyloid deposition and Tau protein fibrillary tangles together contribute to neuronal degeneration. In addition, insoluble Aβ protein is deposited in the intracerebral vessels, making them narrow and less elastic, etc., constituting CAA (right).

AD is a prevalent neurodegenerative disease characterized by extensive brain atrophy and cognitive function deterioration. This progressive condition is speculated to initiate decades prior to symptom onset, frequently without apparent cause (Beason-Held et al., 2013). Epidemiological and genome-wide association studies have implicated ApoE4 as the largest genetic risk factor for late-onset AD (Corder et al., 1993; Shi et al., 2012). ApoE is a lipid transporter, a 299-amino acid protein with a molecular weight of 34 kDa (Sullivan et al., 1998; Guo et al., 2020). It consists of three common subtypes, E2, E3, and E4 (Figure 1), which are similar at the main sequence level but differ in individual amino acid substitution. The negative effects of E4 arise from “domain interactions,” including: the amino-terminal domain containing the LDL receptor-binding region and the carboxyl-terminal domain containing the lipid-binding region, which are held together by a flexible hinge region to form a more compact structure. ApoE is secreted in cells and then lipidated by ATP-bound cassette transporters A1 (ABCA1) and ABCG1, resulting in lipoprotein particles (Nikoulin and Curtiss, 1998; Bu, 2009; Huang, 2010; Yamazaki et al., 2019). Apolipoproteins play a key role in redistributing cholesterol and other lipids to neurons by binding to apolipoprotein receptors on the cell surface. The degree of lipidation of ApoE4 affects its ability to bind LDLR (Huang and Mucke, 2012; Martens et al., 2022). Apolipoproteins have also been found to play a key role in the transfer of lipids from intracellular to extracellular space (Mody and MacDonald, 1995). After neuronal degeneration, apolipoproteins are able to bind lipids and facilitate their redistribution for cell lipid diffusion, membrane repair, or remodeling of new axons. ApoE4 produced in damaged neurons is associated with mitochondrial dysfunction and the formation of NFTs, while ApoE4 produced in astrocytes may be primarily responsible for the formation of Aβ. ApoE4 produced in or around blood vessels is also thought to be important for the formation of cerebral amyloid angiopathy (CAA), and these findings may be particularly important for reducing the risk of AD (Xu et al., 2006; Takeda et al., 2010; Huang and Mucke, 2012; Figure 2). AD is distinguished by two primary pathological hallmarks: extracellular Aβ plaques, which are aggregates of Aβ peptides, also known as senile plaques, and NFTs, which consist of hyperphosphorylated Tau protein within neurons (Koutsodendris et al., 2022). The involvement of chromosome 19 in late-onset AD was previously substantiated by the discovery of ApoE on chromosome 19 and the association of AD with this chromosome (Pericak-Vance et al., 1988, 1991; Strittmatter et al., 1993a). Genetic components have long been associated with AD due to the familial clustering of AD cases (El Haj et al., 2015). In recent years, numerous researches have been carried out on the structural properties, biochemical and pathophysiological effects of neuronal ApoE4. The aim is to reveal the magnitude of the toxic effect of neuronal ApoE4 compared to its traditionally roles of astrocytes and microglia-derived ApoE4. This includes the expression of ApoE4 in neurons, the mechanism of action, and potential treatment options. This review discusses neuronal-derived ApoE4 and possible therapeutic options for ApoE4 from this source to facilitate the development of AD disease research.

## 2. Neuronal ApoE4: production, distribution, and functional role

### 2.1. Expression and distribution of neuronal ApoE4

ApoE is primarily produced by astrocytes under normal physiological conditions in the brain; however, it is also produced by neurons under stress (Konings et al., 2021). However, it is not clear whether ApoE is biologically active with respect to specific cellular sources. Therefore, researchers induced excitatory damage by systemic red alginate injection in transgenic ApoE knockout mice expressing human ApoE isoforms in astrocytes or neurons. ApoE3 expression is protective for neuronal synaptic and dendritic excitation, regardless of its cellular origins. *In vivo* astrocyte-derived ApoE4 has the same protective effect as ApoE3. In contrast, neuronal ApoE4 expression is not only unprotective but also leads to excitotoxicity in cortical neurons, suggesting that neuronal ApoE4 promotes excitotoxic cell death (Buttini et al., 2010). ApoE is most expressed in the peripheral liver and midbrain. There are significant differences in the expression and distribution of ApoE mRNA on different types of hippocampal neuronal cells, and this difference seen in mice is age-related (Chen et al., 2021). Specifically, in mouse gyrus granule cells and CA1 vertebral cells, subtype *ApoE4* gene expression levels increase rapidly at 5 months of age, peak at about 10 months of age, and then begin to decline rapidly with age, dropping to a minimum level at approximately 20 months of age (Bilheimer, 1988); Expression of another subtype of ApoE, ApoE3, tends more slowly, reaching its highest level at about 15 months of age and then rapidly declining (Price et al., 1998); In growth hormone inhibitors/microhyalin interneurons, ApoE4 and ApoE3 expression tends to decrease first and then increase, ApoE3 expression decreases to its lowest level at monthly age, and this decline is faster and greater for ApoE4-KI (Marais, 2019). Researchers have demonstrated that C/EBPβ regulates the expression of *ApoE* gene as a key transcription factor under physiological and pathological conditions, especially the selective upregulation of ApoE4 mRNA expression in neurons (Ruiz et al., 2005). Together, these data suggest that neuronal ApoE, particularly the subtype ApoE4, play a pathogenic role in AD-associated selective neuronal degeneration and loss, age and genetic subtype-related variation in ApoE expression in astrocytes is absent (Bilheimer, 1988; Price et al., 1998; Bilousova et al., 2019; Marais, 2019). In addition, neuronal taxa (excitatory and inhibitory neurons) with high ApoE expression are strongly associated with pre-disease (i.e., mild cognitive impairment stage), while this correlation decreases after disease (i.e., AD dementia stage), and as the disease progresses, a large number of ApoE-expressing neurons decrease with disease progression (Ignatius et al., 1986).

### 2.2. Function of neuronal ApoE4

The ability of neurons to synthesize ApoE is constrained by ApoE immunoreactivity in cortical and hippocampal neurons of AD patients and the elderly For example: entorhinal cortex lesions (ECL) can damage the penetrating pathway in the hippocampus, causing a rapid increase in ApoE mRNA expression in the hippocampus (Poirier et al., 1991; Gómez-Ramos et al., 2001); ApoE binds to esterified cholesterol via the low-density lipoprotein receptor-associated protein (LPR) pathway after perceived entry into neurons (Holtzman et al., 1995). There is also evidence that ApoE originates from neurons, and in one study, ApoE mRNA was also transcribed and expressed in certain neurons in the frontal cortex and hippocampus, but not in cerebellar cortex of the same brain. This region-specific human ApoE gene expression suggests that it probably play a role in the regional vulnerability of AD neurons (Lin et al., 1986; Xu et al., 1999). ApoE, which binds to Apolipoprotein A-I (ApoA-I) and LDL receptors, is involved in a well-established lipid transfer system, raising the possibility that ApoE is also involved in lipid transport that damages nerves (Boyles et al., 1989). And the lipid distribution is different at each stage. In the axon regeneration stage, its tip is rich in LDL receptors; after axon regeneration, ApoE and ApoA-I accumulate distal to the injury site; at this stage of regeneration, ApoE and ApoA-I are present in the extracellular matrix as components of cholesterol-rich lipoproteins (Müller et al., 1985; Geffin et al., 2017). Neuronal ApoE4 is more neurotoxic than ApoE4 secreted by astrocytes. Neuronal ApoE4 causes the onset of AD through a variety of pathophysiological mechanisms, including: stimulating microglia to induce neuroinflammation; directly leads to neuronal degeneration; participating in other signaling pathways mediating the production and aggregation of Aβ and Tau; preventing the excretion of lipids, causing lipid accumulation in cells, etc.; It can also reduce the formation of myelin sheaths by affecting the cholesterol transport of neurons, thus Induce AD pathology (Belloy et al., 2019; Blanchard et al., 2022; Dolgin, 2022). The specific role of ApoE4 in neurons has been further supported by studies showing that selective deletion of ApoE4 in GABAergic interneurons is sufficient to prevent GABAergic interneuron loss and learning and memory deficits in ApoE4 knock-in (KI) mice, whereas astrocyte production of ApoE4 deletion is not protective (Nuriel et al., 2017). The greatest difference in gene expression between the two isoforms was observed when ApoE3 was exposed to HIV cultures along with ApoE4 (Becker et al., 2015). HIV exposure amplified the inhibitory effect of ApoE4 on neuronal gene expression. This neuron-specific response to ApoE4 may affect neurogenesis and neuronal survival (Becker et al., 2015; Geffin et al., 2017).

## 3. Neuronal ApoE4 mediates AD pathogenesis

### 3.1. Mechanisms of neuronal ApoE4 on Aβ aggregation

Previously, the mainstream theory agreed that Aβ was mainly deposited outside neurons, but the latest study found that in the early stage of AD disease, Aβ mainly accumulates inside neurons; And the detection of deposited Aβ plaques found that the plaques were (poison flower) plaques (i.e., the remains of neurons) formed after the death of neurons after autophagy disorders (Long and Holtzman, 2019). Studies in humans and transgenic mice have shown that brain Aβ levels and amyloid plaque loading ApoE subtype dependence, in order, are (ε4 > ε3 > ε2; Zalocusky et al., 2021). ApoE can interact directly with Aβ to form stable complexes (Mahley et al., 2009), also seen in senile plaques and NFTs (Najm et al., 2020). The colocalization of major neuropathological features of ApoE with AD coupled with the enrichment of the E4 allele suggests a relationship with the etiology of AD (Figure 2). For example, the presence of the E4 allele in susceptible individuals may exacerbate the age-related decline in cell numbers and lipid content that typically occurs in the human brain (Mahley et al., 2009; Najm et al., 2020; Taubes et al., 2021; Zalocusky et al., 2021). In addition, the *in vivo*-lysosomal pathway is thought to have a regulatory role in the pathophysiology of AD, as alterations in this pathway can damage leading to the accumulation of harmful proteins (Xia et al., 2021). ApoE4 leads to functional defects within neurons through lysosomal degradation pathways in dysregulated bodies (Parhizkar and Holtzman, 2022). *In vivo* studies using ApoE/hAPPFAD ApoE4 resulted in a significant increase in the number of Aβ deposition and neuritis plaques in mice compared to ApoE3 (Chen et al., 2022). Analysis of postmortem AD tissue showed that ApoE4 was also involved in the formation and aggregation deposition of new plaques, and that the carrier of ApoE4 had a higher ApoE-Aβ complex deposition capacity compared to non-carriers (Najm et al., 2020; Chen et al., 2022; Lee et al., 2022). Aβ polymerization is the process by which soluble Aβ peptides undergo conformational changes to form insoluble fibers (Wang et al., 2021). ApoE acts as a pathological carrier, promoting the conformational changes of Aβ protein from soluble to insoluble fibrils. E4 is stronger than E3 in promoting the formation of insoluble Aβ proteins (Namba et al., 1991; Strittmatter et al., 1993b; Verghese et al., 2011; Van Acker et al., 2019). In addition, CSF Aβ_42_ levels and PiB imaging in ApoE4 carriers, both of which suggest cerebrospinal fluid amyloid deposition (Miranda et al., 2022). Cognitively normal patients with ApoE4 show PiB-positive imaging around age 56 years, while noncarriers show PiB-positive imaging around age 76 (Holtzman et al., 2000). The lipid-binding fragment of ApoE4 works synergistically with the receptor-binding region, resulting in mitochondrial dysfunction and neurotoxicity, which can contribute to the onset of AD (Kloske and Wilcock, 2020).

### 3.2. Neuronal ApoE4 mediates tau aggregation

The MAPT gene on the long arm of chromosome 17 encodes Tau protein, whose function in the normal brain is to bind to tubulin to promote its polymerization to form microtubules; when Tau protein is abnormally over phosphorylated, the binding force with tubulin will be greatly reduced, losing its biological function of promoting microtubule assembly and losing its role in maintaining microtubule stability (Kanekiyo et al., 2014). Selectively splicing to the N-terminal domain (N), microtubules combined to the repeating domain (R) to form six different isoforms (0 N3 R, 0 N4 R, 1 N3 R, 1 N4 R, 2 N 3R, and 2 N 4 R), which are expressed differently during brain development (Castano et al., 1995). Some studies suggest that aggregation and hyperphosphorylation of Tau proteins may be important drivers of AD neurodegeneration (Kok et al., 2009). ApoE regulates Tau-related pathology and Tau neurodegeneration (Xiong et al., 2021). In cognitively functioning ApoE4 carriers, Functional magnetic resonance imaging (fMRI) showed an increase in signal intensity during memory activation function and increase in the number of activated regions in the hippocampus. Together, these data suggest that ApoE4 may cause neuronal dysfunction in two ways: directly leading to neurotoxic effects and promoting overactivity of neuronal hippocampal networks (Schmechel et al., 1993). Compared to E3/E3, neurons with E4/E4 iPSCs have more hyperphosphorylated Tau and fewer GABAergic neurons (Castano et al., 1995; Kok et al., 2009; Reiman et al., 2009; Kloske and Wilcock, 2020; Xiong et al., 2021). Summarizing *in vivo* and *in vitro* data, ApoE3 and ApoE4 have different effects on Tau phosphorylation and aggregation. ApoE3 forms a stable complex with Tau *in vitro*, while ApoE 4 does the opposite (Prince et al., 2004). The expression of human ApoE4 transgenes in mouse neurons found an increase in phosphorylation of Tau, but the expression of human ApoE4 in mouse astrocytes did not increase phosphorylation of Tau (Vidal et al., 2000; Chang et al., 2005) indicating that ApoE4 can specifically affect the phosphorylation of Tau protein in neurons (Sato et al., 2018). Higher levels of zinc in brain tissue in AD patients increase ERK1/2 activation, leading to increased ApoE4-induced Tau phosphorylation (Sayed et al., 2018). In addition, AD-related oxidative stress due to folate and vitamin E deficiency can also increase Tau phosphorylation of ApoE4, which can be prevented by supplementation with S-adenosylmethionine (Vaquer-Alicea et al., 2021). The C-terminal fragment of ApoE truncated can damage neuronal cells *in vitro*, resulting in the formation of inclusion bodies in the cytoplasm of some cells (Najm et al., 2019). Thus, ApoE 4 fragment-induced neurotoxicity may be associated with cytoskeletal disruption (Li et al., 2016).

### 3.3. Neuronal ApoE4 leads to neuroinflammation

Neuroinflammation is an important central mechanism involved in neuronal functional degeneration. Neuronal ApoE4 can lead to associated neuroinflammation, thereby increasing the prevalence of AD (Strittmatter et al., 1994; Brecht et al., 2004). Mice with ApoE4 are injected with lipopolysaccharides (LPS) peripherally and initiate immunity, producing elevated levels of pro-inflammatory cytokines (e.g., TNFα, IL-6) and injury-induced nitric oxide (Tesseur et al., 2000a,b). In glial cell culture, the presence of ApoE contributes to inhibition of glial cell activation of LPS, suggesting that ApoE provides a protective anti-inflammatory response (Richey et al., 1995; Harris et al., 2004; Chan et al., 2009). With the beginning of animal studies, researchers have begun to study how ApoE and its subtypes cause neuroinflammation (Poirier, 2000; Huang, 2011; Uddin et al., 2019). In a previous study, long-term administration of LPS to APP mice with and without ApoE showed increased glial hyperplasia and Aβ deposition, suggesting that ApoE has an increased inflammatory effect in AD models (Uddin et al., 2019). Another model study using human ApoE3 and ApoE4 showed that the ApoE subtype plays a regulatory role in the production of nitric oxide (NO) by microglia (Vitek et al., 2009). They found that ApoE4-positive microglia release significantly more NO than ApoE3 microglia, which may lead to Aβ inducing neuronal death by activating astrocytes (Lynch et al., 2003). Reactive “microglial hyperplasia,” manifested by morphological abnormalities (e.g., from branching morphology to amoeba morphology), increased immunoreactivity of ionized calcium-binding bridging molecule 1 (IBA1), and increased proliferation, are a distinctive feature of AD pathology, particularly around amyloid plaques in postmortem brain tissue (Laskowitz et al., 1998). Some studies suggest that members of the TLR family and CD14 are membrane receptors which responsible for activating the immune system and are involved in mediating inflammatory activation of Aβ by astrocytes and microglia (Laskowitz et al., 1997). Blocking TLR2 or TLR4 with specific antibodies reduces microglial activation after Aβ peptide excitation (Barger and Harmon, 1997; Pocivavsek et al., 2009). Microglia that knock down TLR2 and TLR4 also show a reduced inflammatory response to Aβ (Bales et al., 2000; Qiao et al., 2001). However, the extent and specific mechanisms of the link between neuroinflammation and cognitive dysfunction in AD patients are not fully understood. In contrast, inflammatory markers were associated with cognitive decline in transgenic mouse models (Dorey et al., 2014) and patients (Colton et al., 2002), suggesting that neuroinflammation is a potentially attractive therapeutic target for AD patients.

## 4. Potential strategies for targeting neuronal ApoE4 in the treatment of AD

### 4.1. Regulation of ApoE4 lipidation and quantity

The lipidation of ApoE *in vivo* is mainly carried out by ABCA 1 in the brain (Dorey et al., 2017; Bisht et al., 2018). MicroRNA-33 (miR-33) has been shown to regulate ABCA 1 levels and ApoE lipidation in the brain, and antisense oligonucleotides specifically inhibit miR-33 in the brain, significantly reducing Aβ plaques in the cortex (Jana et al., 2008; Kim et al., 2015). In addition, ApoE has two main metabolic receptors: LDLR and LRP, which facilitate lipid transport between different cell types (Udan et al., 2008). Given that ApoE expression is controlled by peroxisome proliferators-activated receptor γ and LXR (complex with RXR), agonists or antagonists of these nuclear receptors are potential candidates for ApoE pathway modulators (Erridge et al., 2008). In fact, recent studies have shown that oral administration of RXR agonist (bexarodonene) in amyloid mouse models reduces the aggregation and deposition of Aβ plaques and enhances cognitive function in mice in an ApoE-dependent manner (Liu et al., 2012). In mouse amyloid models, the LXR agonist TO901317 has also been shown to increase ApoE levels in the brain, promote clearance of Aβ_42_, and reverse memory deficits (Chawla et al., 2001). In addition to ApoE, LXR regulates ABCA 1 to promote cholesterol excretion. It has been shown that the increased tendency for ApoE4 self-aggregation reduces the recycling of ABCA-1 membranes, resulting in decreased lipidation of ApoE4 particles (Kim et al., 2015). Targeted lipidomics analysis by knocking ApoE into mice showed that ApoE4 mice increased the sensitivity of the Entorhinal cortex (EC) to lipid changes (Kim et al., 2009). Regarding the lipid-binding capacity of ApoE, ApoE4 showed binding preference for both VLDL and LDL, while apoE3 showed higher affinity for HDL (Hirsch-Reinshagen et al., 2005). ApoE3 and ApoE2 have a higher binding affinity for HDL than ApoE4, which has a stronger binding affinity for VLDL (Cramer et al., 2012). This suggests that upregulation of lipid ApoE may be required to maximize the therapeutic efficacy of AD.

In addition, altered levels of ApoE expression serve as underlying disease-modifying therapies, particularly the amount of ApoE4, which largely determines the deposition and clearance of Aβ (Vanmierlo et al., 2011). It has been found that simply knocking down APOE4 (e.g., cre-mediated ApoE4 excision or creating an ApoE4 haploid model) reduce levels of Aβ plaques deposited in the brains of APP transgenic mice (Rawat et al., 2019),(Kunzler et al., 2014). Topical direct application of anti-ApoE antibodies into the brain prevents Aβ plaque deposition forming and reduces pre-existing plaques. Among several current monoclonal anti-ApoE antibodies, HJ6.3 antibodies significantly reduce amyloid deposition by 60–80% after 14 weeks of injection (Raffai et al., 2001), antibody therapy can work synergistically with anti-Aβ immunotherapy to achieve maximum reduction of Aβ plaques (Nguyen et al., 2010). The researchers report that in the APP/ApoE4 mouse model, antibodies that preferentially identify non-lipidated forms of ApoE4/ApoE3 over lipidated forms such as “HAE-4” are highly effective in preventing Aβ deposition through FcγR-dependent mechanism (Kim et al., 2011). Several research groups have now adopted antisense knockout techniques, which use antisense oligonucleotides (ASOs) as therapeutic agents to inhibit protein synthesis and reduce ApoE levels. The results showed that the drug was well tolerated by the participants, and the most common side effect was headache after injecting the drug, but there were no serious adverse events in the treatment group. This suggests that the use of ASO as a first-line treatment agent for a range of neurodegenerative diseases may have great potential. Knocking APP PS 1–21 × ApoE3 and APP PS 1–21 × ApoE4 into mice and injecting antisense oligonucleotides into the ventricles at birth halved soluble ApoE concentrations, resulting in lower soluble and insoluble Aβ levels, resulting in a decrease in dense central plaques, but these Aβ measurements remain virtually unchanged when Aβ plaque deposition begins. Regarding the targeting of ASO ApoE expression, efforts are still in preclinical testing. It is worth noting that ASO did not show this side effect compared to ApoE receptor agonists, which caused adverse reactions of the lipid metabolism system. ASO therapy has the potential to become a future treatment for AD patients (Liao et al., 2018; Table 1).

**Table 1:** Potential therapeutic approaches for targeting APOE.

	Potential methods	Function	Limitations
Target ApoE esterification/quantity	MircoRNA-33 anti-oligosensitive nucleotide (Chawla et al., 2001; Kim et al., 2009, 2015)	Regulates ABCA1 expression and esterification levels, increases response to ApoE transport	Efficient delivery to a variety of organizations
	LDLR/LRP (Hirsch-Reinshagen et al., 2005)	Carries lipids	Lack of receptors
	Peroxisome-activated receptor r and LXR agonist (T0901317; Cramer et al., 2012; Vanmierlo et al., 2011)	Regulates apolipoprotein expression	Significantly toxic
	Establishment of ApoE4 haploid model (Kim et al., 2011, 2012)	Reduces ApoE4 levels and upregulates apolipoprotein	Inefficient build/reorganization
	HJ 6.3 antibodies (Liao et al., 2018; Bennett, 2019; Williams et al., 2020)	Anti-ApoE antibodies	Immunotherapy carries a risk of adverse immune response,
	ASO (Huynh et al., 2017; Lanfranco et al., 2020; Serrano-Pozo et al., 2021)	Inhibits protein synthesis and reduces apolipoprotein levels	Headache after injecting the drug
Target ApoE structure/association with Aβ	GIND25 (Fryer et al., 2005; Ramaswamy et al., 2005; Mahley et al., 2006; Huang and Mahley, 2014; Li et al., 2022)	“Structural aligners” Destruction of the ApoE4 domain	The *in vivo* effect of structural correctives has not been examined
	Aβ12-28P peptide (Sadowski et al., 2004; Deroo et al., 2015; Hu et al., 2015; Wisniewski and Drummond, 2020)	Destruction of ApoE-Aβ structure	BBB penetration is limited
	Congo Red (CR; Sawmiller et al., 2019)	Inhibits Aβ self-binding and interacts with Aβ in the same region as apolipoprotein	Carcinogenic
	Peptide 6KApoEp (DeMattos et al., 2001; Sawmiller et al., 2019)	Inhibit the binding of ApoE to the N-terminal of the APP	There are many factors that affect the role of ApoE
	Monoclonal antibody M266 (Leissring, 2008)	Alters the central system and clearance of plasma Aβ, reduces the burden of Aβ in the brain	Clinical trials were not successful
	Aβ hydrolase (Turner and Nalivaeva, 2007; Holtzman et al., 2012)	NEP, insulin-degrading enzyme, angiotensin-converting enzyme, serous protease system	The enzyme environment is strict

### 4.2. Targeting ApoE’s structural properties as well as interactions with Aβ

ApoE4 is structurally different from ApoE2 and ApoE3 due to differences in interactions between ApoE domains, and this difference can lead to harmful effects specific to ApoE4 subtypes. Therefore, altering the structure of ApoE4 to form ApoE3-like molecules may be an interesting way to improve these harmful effects (Lanfranco et al., 2020). The domain interaction properties of ApoE4 reduces its secretion from cells (Ramaswamy et al., 2005) while making it unstable against proteases (Mahley et al., 2006), leading to pathogenic effects (Chen et al., 2011). Therefore, another potential treatment is to disrupt this ApoE4 domain interaction with a “structural corrector” and treat ApoE4-expressing Neuro-2a cells with this structural corrector to make the protein more “ApoE3-like” in structure and function (Huang and Mahley, 2014). Other effective small molecules that inhibit interdomain interactions and thereby reverse the toxic effects of ApoE4 have been identified using a high-throughput FRET preliminary screening assay (to measure changes between ApoE4 domain interactions) and a set of neuronal cell-based secondary functional assays. A computer screen was used to identify the prototype structural corrector GIND-25 (Ye et al., 2005). Treating neurons with GIND-25 reduces the production of ApoE4-stimulated Aβ (Fryer et al., 2005; Liu et al., 2013) and restores mtCOX 1 levels in ApoE4-expressing cells (Fryer et al., 2005). This destructive effect of structural correctors effectively reduces Aβ aggregation and deposition and can be used as a reliable treatment (Li et al., 2022). Once considered incurable, PPI inhibitors have become an irreplaceable drug due to a significantly improved understanding of the chemistry of PPI stents (Ran and Gestwicki, 2018). Another inhibitor of ApoE-Aβ is a peptide mimic called Aβ12-28P, because the binding of APOE to Aβ occurs at positions 244–272 residues of ApoE and 12–28 residues of Aβ (Pankiewicz et al., 2014), Aβ12-28P peptide targeting this site is able to cause reduced Aβ oligomerization and plaque deposition in ApoE2-targeting (TR) or ApoE4-TR amyloid Tg mice (Hu et al., 2015), which confirms inhibition of Aβ/ApoE interactions are therapeutically beneficial. It is a fibril-forming and nontoxic Aβ derivative with Aβ12-28P consistent with BBB permeability (Sadowski et al., 2004). In cell culture, the peptide reduces the neurotoxicity of Aβ by preventing the binding of ApoE and Aβ at residues 12–28. Furthermore, the peptide is known to bind to other peptides (Deroo et al., 2015; Ghosh et al., 2019). The Aβ residue between 17 and 21 appears to be a key region for Aβ to bind to ApoE, and lysine for residue 16 is particularly important because peptides that conform to this sequence effectively inhibit Aβ/ApoE binding (Liu et al., 2011). Disruption of ApoE-Aβ interaction with Aβ12-28P significantly reduces the amount of Aβ in neurons and inhibits the loss of synaptic proteins in this co-culture system (Kuszczyk et al., 2013). Notably, these improvements were not due to immunity, as these mice did not produce antibodies against this Aβ fragment. The efficacy of Aβ12-28P has not been demonstrated in clinical trials (Liu et al., 2014; Wisniewski and Drummond, 2020). In addition, Congo red (CR) effectively bind to fibrils and early aggregates. Notably, CR has been shown to inhibit self-binding of Aβ and interact with Aβ through the exact same region as ApoE, meaning that CR can block ApoE/Aβ binding (Carter and Chou, 1998). A new inhibitor of ApoE-Aβ interaction, called peptide 6KApoEp, inhibits ApoE binding to the N-terminus of the APP (Sawmiller et al., 2019). In transgenic mouse models of AD, immunotherapy against Aβ can also reduce Aβ deposition. In active immunity, monoclonal antibodies (m266) against the Aβ central domain have been found to reduce brain Aβ burden by altering CNS and plasma Aβ clearance (DeMattos et al., 2001). Aβ hydrolase is a reliable treatment for AD with a good reduction of the number of Aβ, including the enkephalin (NEP) family of metalloproteases (including its homogeneous endothelin converting enzyme), insulin-degrading enzyme, angiotensin-converting enzyme, plasmin, and possibly some other enzymes, which are degraded from the brain by proteolytic degradation (Turner and Nalivaeva, 2007; Leissring, 2008; Table 1). The above results suggest that ApoE-Aβ interaction inhibitors have the potential to be used to treat Aβ and Tau load that reduce central nervous system levels.

### 4.3. Targeting ApoE receptors

LRP 1 is a member of the LDL receptor family, LDL receptor-associated protein (LPR 1), LDL-associated protein 1B (LRP 1B), LRP 2, VLDL receptor, ApoE receptor 2 (apolipoprotein 2/LRP 8), LRP 4/MEGF 7, and more distant members LRP 5, LRP 6, and protein-associated receptor sorting class A LDLR repeats (SorLA/LR; Dodart et al., 2005; Lillis et al., 2008; Holtzman et al., 2012). LRP 1 is one of the larger members of the LDL receptor family, approximately 600 kDa in size (Herz and Strickland, 2001). Ligands for LRP1 include the AD-associated proteins ApoE, APP, Aβ, and macroglobulin α2 (α2M), tissue plasminogen activating protein (tPA), and APP (Martiskainen et al., 2013; Lane-Donovan and Herz, 2017). Aβ clearance mechanisms associated with LRP 1 include intracellular degradation, via BBB efflux cycle, and peripheral clearance. ApoE binds primarily to LDLR family receptors (Herz and Bock, 2002),(Herz and Chen, 2006). LDLR binds LDLs containing Apolipoprotein B (ApoB) and ApoE particles based on density through electrostatic interactions between basic amino acid residues and acid residues on ApoB/ApoE (Katsouri and Georgopoulos, 2011). Like LDLR, LRP1 undergoes receptor-mediated endocytosis and transports ligands from the cell surface into the cell; However, the endocytosis efficiency of LRP1 is higher than that of LDLR (Hussain, 2001). Although there are certain differences in the brain, both play an important role in ApoE metabolism. First, LRP1 is highly expressed in neurons and less in glial cells, while LDLR is the opposite (Rebeck et al., 1993; Levi et al., 2005). Kounnas et al. demonstrated that LRP 1 binds to Kunitz-containing eggs outside the cyto-soluble APP of the leukocyte inhibitor (KPI) domain (APP 751 and APP 770) and mediates their cellular catabolism (Kounnas et al., 1995; Trommsdorff et al., 1998; Pietrzik et al., 2004). The result of the interaction between APP and LRP1 is that APP endocytosis transports and processes to Aβ acceleration (Pohlkamp et al., 2017; Table 2). There is considerable indirect evidence that ApoE receptors are involved in APP metabolism and kinase phosphorylation. In addition, lifestyle changes may increase the risk of developing AD in ApoE4. This lifestyle change includes dieting and exercise. Results from human clinical trials are mixed, but overall, the amount and type of exercise beneficial for AD needs to be determined. A large number of clinical trials are currently being recruited to study the type of exercise and regimen that would be most beneficial for AD (Troutwine et al., 2022).

**Table 2:** LDLR receptor family species and corresponding functions.

Receptor	Function
LDLR	Binding lipidized ApoE particles (Bilheimer, 1988; Nikoulin and Curtiss, 1998; Bu, 2009; Marais, 2019)
VLDLR	Bind non-lipidated ApoE (Price et al., 1998; Bilousova et al., 2019; Marais, 2019)
LRP1	Bind to recombinant ApoE or ApoE aggregates (Bilheimer, 1988; Buttini et al., 2010; Marais, 2019)
LRP2/Giant proteins	Endocytic receptors, reuptake of multiple ligands (Herz and Bock, 2002; Lane-Donovan and Herz, 2017)
LRP1B	Cell proliferation (Castano et al., 1995; Lillis et al., 2008)
LRP5/LRP6	A core member of the LDLR family, normalized Wnt (Kanekiyo et al., 2014)
SorLA/SorL1	Intracellular protein trafficking (Herz and Strickland, 2001; Dodart et al., 2005; Lillis et al., 2008)
ApoER2	Mediates endocytosis and participates in the Reelin signaling pathway (Castano et al., 1995; Kounnas et al., 1995)
HSPGs	Binding of esterified and non-esterified ApoE on the cell membrane surface (Rebeck et al., 1993; Ruiz et al., 2005)

## 5. Discussion

Many studies on AD have shown that ApoE play an important role in the hereditary pathogenesis of AD. The latest research shows that when neurons are stimulated by aging, damage and other factors, it becomes the main source of ApoE4, and its neurotoxicity is much greater than that secreted by astrocytes. It has been found that neuronal ApoE4 mediates the pathophysiology of AD may include: prevents lipid excretion; induces inflammation of microglia to damage neurons; participation in the occurrence of Tau pathology, etc.; and studies have confirmed that selective removal of ApoE4 from neurons significantly reduce Tau pathology, glial proliferation, neurodegeneration, neuronal hyperexcitability, and myelin deficiency (Koutsodendris et al., 2023). However, the specific mechanism has not been fully explained. As well as another pathological feature of AD: Aβ plaques, it is still unknown whether neuronal ApoE4 mediates the formation of Aβ and the specific pathophysiological mechanism. Several potential treatment options for AD targeting ApoE4 also been collated and discussed. At present, drugs in terms of ApoE4 quantity and lipidation are mainly agonists of liver X receptor (LXR) and retinol X receptor (RXR)**-**Bexarotene is approved for the treatment of cutaneous T-cell lymphoma and can be reused for other indications. It has been used in the treatment of non-small cell lung cancer, breast cancer, and Kaposi sarcoma (Farol and Hymes, 2004). Bexarotene has been observed to be effective in alleviating pathological and cognitive deficits in animals with Animals with Aβ pathology, but the use of bexarotene in AD is controversial due to its significant toxicity, typically increasing triglyceride and cholesterol levels and increasing the risk of hypothyroidism (Graeppi-Dulac et al., 2014; Cummings et al., 2016). Another treatment that targets the regulation of ApoE4 lipidation, the small molecule miR-33, whose inhibition increases ABCA1 expression and activity in rodents and non-human primates, thereby increasing ApoE4 lipidation without serious metabolic adverse reactions (Jan et al., 2015). The deficiency of miR-33 also increases HDL-C and increases the outflow of cholesterol from macrophages through ABCA 1 and ABCG 1, which prevents the progression of atherosclerosis (Horie et al., 2012). The above conclusions have been supported by experimental data, but the disadvantage is that many genes are changed after knocking down miR-33 in mice, so detailed experiments are needed to establish miR-33-targeted therapies in humans. Active Aβ_42_ immunity in AD patients leads to Aβ removal, associated with a decrease in phosphorylated Tau, long-term downregulation of inflammation, a decrease in the number of neurons, and an abnormal decrease in neuritis (Nicoll et al., 2006; Paquet et al., 2018). Active immunity with Aβ carries a risk of adverse immune response, leading to inflammation such as meningoencephalitis, and lacks the ability to regulate the level and duration of the response (Paquet et al., 2018). Passive immunity induces antibody–antigen complexes that are fully ligated to the Fc γ receptor (Fc γ R) on microglia, which can cause adverse pro-inflammatory reactions that may lead to observed BBB disruption such as angiogenic edema and/or cerebral microhemorrhage. In contrast, passive immunity may provide a safer treatment option for AD immunotherapy (Orgogozo et al., 2003; Adolfsson et al., 2012). Another therapeutic drug ASO, which can be used as therapeutic agents by disrupting the synthesis of specific proteins, are considered as first-line treatments for several neurodegenerative conditions such as polyneuropathy, muscular dystrophy, and spinal muscular atrophy (Hinrich et al., 2016; DeVos et al., 2017; Scoles et al., 2019). ASO therapy led to improved synaptic function in mice with Aβ pathology, and specifically targeting ApoE has been successful in reducing Aβ pathology in APP/PS1 mice when treated before Aβ deposition begins (Paquet et al., 2015). At present, the expression of microtubule-associated protein Tau by ASO-targeting has been subjected to phase Ib clinical trials (Mummery et al., 2023), and the results showed that the subjects are well tolerated by the drug, and the most common side effect is headache after injection, but no serious adverse events have been seen in the patients with the treatment. It is worth mentioning that ASO therapy QALSODY (tofersen) is currently available for the treatment of amyotrophic lateral sclerosis (ALS; Mummery et al., 2023). Moreover, drugs targeting the structural properties of ApoE4, phase IIa clinical trials of structural correctors (VX-809) and phase III trials of synergists (VX-770) have been completed (Chen et al., 2012). Despite the failure of the anti-Aβ monoclonal antibody M266 in clinical trials, aducanumab was recently approved as the first Aβ monoclonal antibody approved for the treatment of AD, bringing new hope to AD patients around the world. For promising treatment options, the efficacy of which can now be monitored with biomarkers, such as new imaging techniques (PET) and CSF, CSF and PET were used in a report on the prevalence of amyloid abnormalities, including APOE carrier status, dementia severity, etc., to estimate the prevalence of amyloid abnormalities (Jansen et al., 2022). Last year, a clinical research team combined plasma p-Tau, ApoE genotype and MRI brain atrophy measurements to accurately predict Aβ PET deposition (Gao et al., 2022). However, problems such as expensive and invasive injury have not been properly addressed in clinical practice (Mahaman et al., 2022). Other promising sources of biomarkers are saliva, tears, and urine (Blennow and Zetterberg, 2018; Mahaman et al., 2022; Teunissen et al., 2022). They have the advantage of being non-invasive, and a lot of research is being done from these. However, the specific therapeutic role still needs to be studied, which includes metabolisms and toxicology, as well as clearer and more accurate pharmacological data.

In summary, many researches have been done on ApoE4 and AD in the past, but they are limited to a certain cell or pathway. People with the ApoE4 gene not only have a high risk of AD, but also have an early onset of AD. and ApoE4 is a risk factor for the onset of AD, rather than a direct causative factor; ApoE4 works in combination with other mechanisms to cause the onset of AD. Therefore, finding the etiology of synergy with ApoE4 and identifying the mechanism is the key to intervention in this risk factor. At present, the study of neuronal ApoE4 has only explored its aggravation of Tau pathology, but how neuronal ApoE4 mediates pathological changes in other cells remains to be proven, and a stable method is found to specifically knock out the ApoE4 gene in neurons to improve pathological symptoms. Future research needs more in-depth and comprehensive research and exploration. It is necessary to know the complete system of neuronal ApoE4 and AD pathophysiology and develop more effective treatment methods. Exploring the mechanism of neuronal ApoE4 and AD is challenging, but it provides new insights into the prevention and treatment of AD.

## Author contributions

LZ collected data and wrote the manuscript. YX and YG proofread and modified the manuscript. All authors contributed to the article and approved the submitted version.

## Funding

This work was supported by grants from the Natural Science Foundation of Hubei Province of China for Distinguished Young Scholars (grant no. 2022CFA104) to YX.

## Conflict of interest

The authors declare that the research was conducted in the absence of any commercial or financial relationships that could be construed as a potential conflict of interest.

## Publisher’s note

All claims expressed in this article are solely those of the authors and do not necessarily represent those of their affiliated organizations, or those of the publisher, the editors and the reviewers. Any product that may be evaluated in this article, or claim that may be made by its manufacturer, is not guaranteed or endorsed by the publisher.
